# Supplementary material for: Induced p53 loss in mouse luminal cells causes clonal expansion and development of mammary tumours
Source: Nat Commun. 2017 Feb 13;8:14431. doi: 10.1038/ncomms14431 (PMC5316831; doi:10.1038/ncomms14431)
Supplement: Supplementary Information — Supplementary Figures, Supplementary Table and Supplementary Reference [file ncomms14431-s1.pdf]

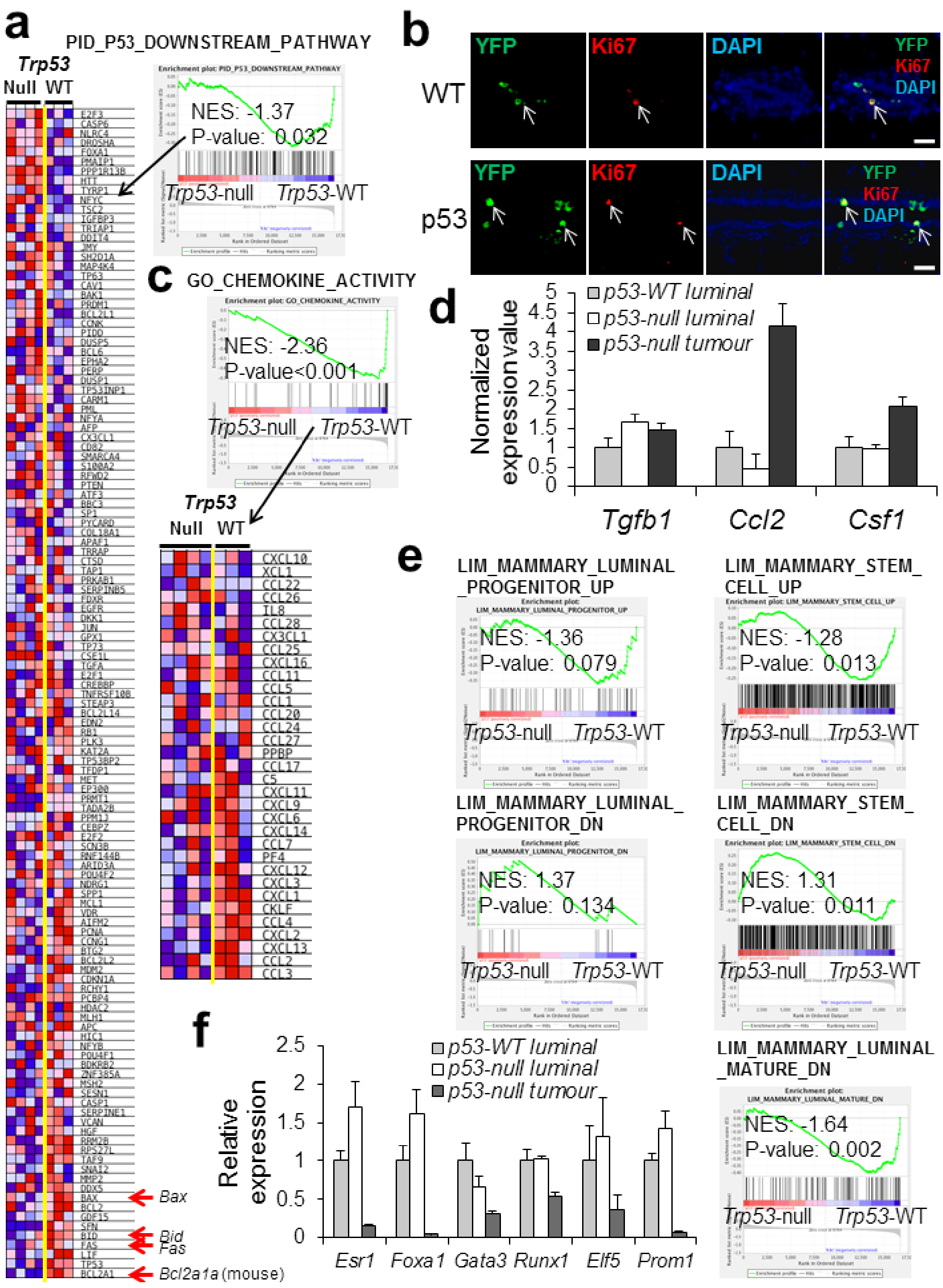

Supplementary Figure 1. Additional data for microarray analysis of *Trp53*-null luminal MECs and validation.

(a) GSEA showing the heatmap and enrichment plot of a gene set related to p53 downstream pathway that is significantly downregulated in *Trp53*-null luminal MECs compared to WT luminal MECs. Note in the heatmap, the top downregulated genes in *Trp53*-null luminal MECs are related to apoptosis control (arrows).

(b) Representative co-IF pictures showing YFP (green) and Ki67 (red) staining for MG sections from *Trp53*$^{L/L}$*;R26Y* females (p53, n=3) or *R26Y*-only females (WT, n=3) 3-4 weeks after *Ad-K8-Cre* injection. YFP^{+} Ki67^{+} cells are indicated by arrows. Scale bar = 20 μm.

(c) GSEA showing the heatmap and enrichment plot of a gene set related to chemokine/cytokine genes that is significantly downregulated in *Trp53*-null luminal MECs compared to WT luminal MECs.

(d) Changes in expression of select cytokine genes in luminal MECs with induced p53 loss; note *Ccl2* was reduced in *Trp53*-null luminal MECs (4 weeks after induced p53 loss) but increased in the resulting *Trp53*-null mammary tumours, *Tgfb1* was increased in both *Trp53*-null luminal MECs and tumours, whereas *Csf1* was only increased in *Trp53*-null mammary tumours.

(e) Additional GSEA results for *Trp53*-null versus WT luminal MECs based on several gene sets related to MEC subset-specific signatures (i.e., conserved signatures from matched subpopulations of human and mouse MECs) from a previous study (Supplementary reference 1).

(f) Relative expression values of select MEC-specific transcription factor and lineage-specific marker genes in *Trp53*-null luminal MECs and mammary tumours in relation to WT luminal MECs (=1), based on microarray expression profiling.

Data represent mean ± SEM. In each enrichment plot, normalized enrichment score (NES) and nominal *P* value (P-value) are shown.

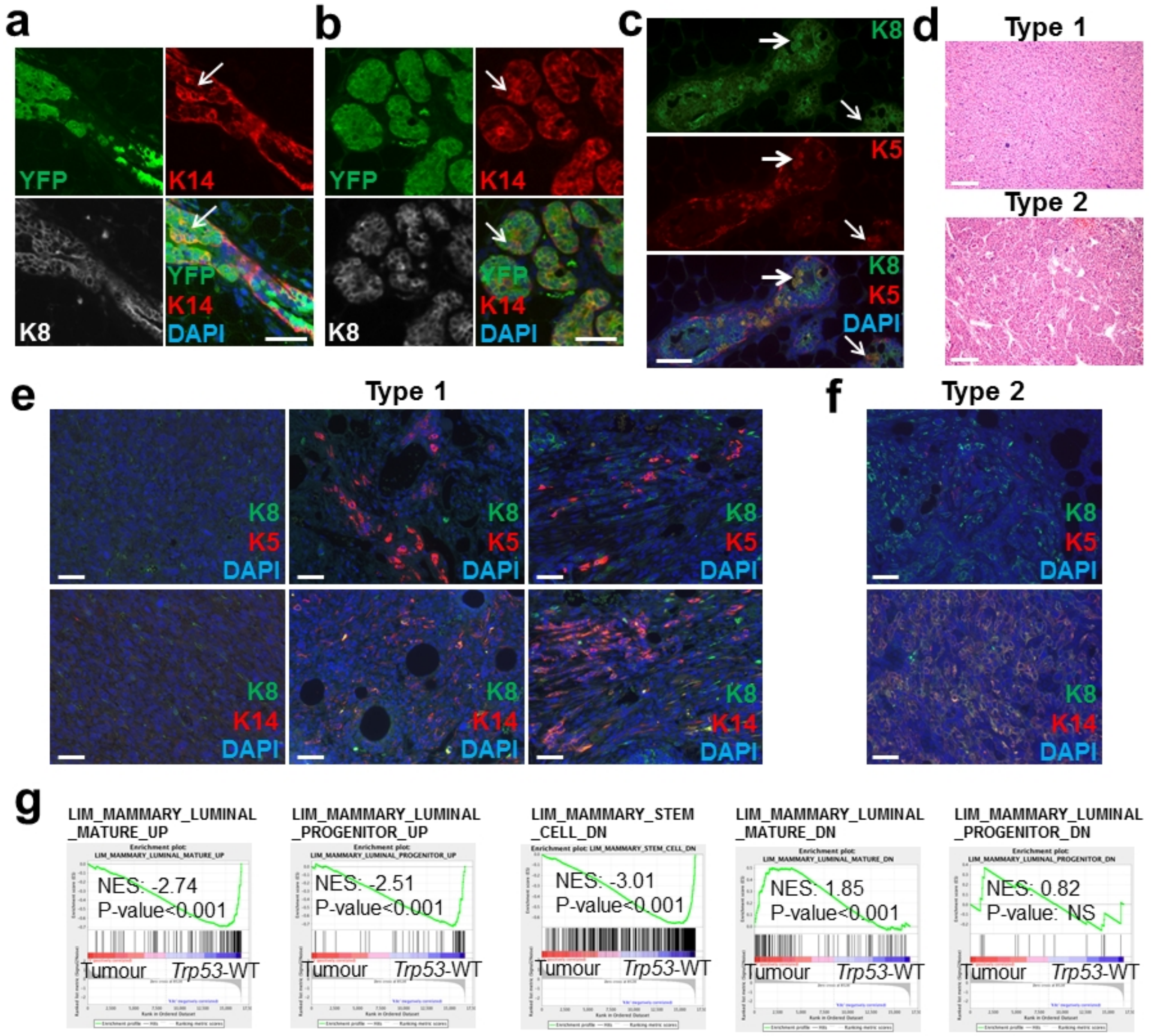

Supplementary Figure 2. Additional data for characterization of *Trp53*-null premalignant lesions and mammary tumours with a luminal origin.

(**a-b**) Co-IF staining showing emergence of abnormal YFP^{+} MECs with basal differentiation (K8^{+}K14^{+} MECs, arrows) in an *in situ* lesion found in the duct (**a**) and an alveolar-like lesion (**b**) from *Trp53$^{L/L}$;R26Y* females >6 months after intraductal injection of *Ad-K8-Cre* adenovirus. Scale bars = 50 µm.

(**c**) Co-IF staining showing emergence of abnormal K8$^+$K5$^+$ MECs (arrows) in premalignant lesions found in a *K8-CreER;Trp53$^{L/L}$* female >6 months after tamoxifen injection. Scale bar = 50 μm.

(**d**) Hematoxylin and eosin (H&E) staining showing morphologies of the two types of mammary tumours developed in female mice with induced loss of p53 in luminal cells. Scale bars = 100 μm.

(**e-f**) Additional co-IF staining pictures showing representative Type 1 [**e**, three tumours with both K8/K5 co-staining (top) and K8/K14 co-staining (bottom)] and Type 2 (**f**) tumours with different levels of K8$^+$ (green), K5$^+$ (red) and K14$^+$ (red) cells. Scale bars = 50 μm.

(**g**) Additional GSEA results for *Trp53*-null mammary tumours compared to *Trp53*-WT luminal MECs (i.e., cells of origin) based on several gene sets related to MEC subset-specific signatures from a previous study (Supplementary reference 1). In each enrichment plot, normalized enrichment score (NES) and nominal *P* value (P-value) are shown.

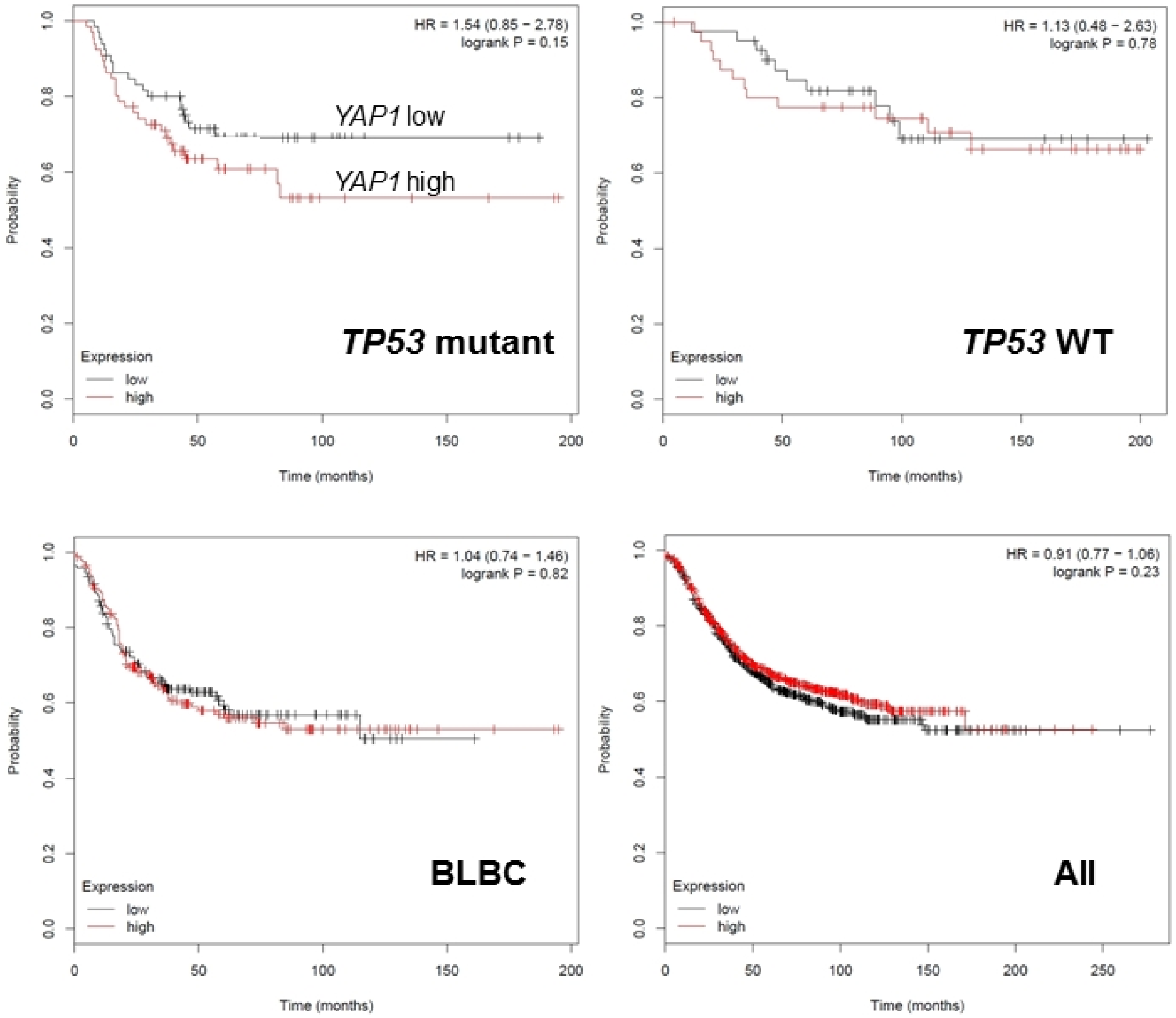

Supplementary Figure 3. Supporting data for SNP analysis of *Trp53*-null mammary tumours with a luminal origin.

Kaplan-Meier plots based on Recurrence-free survival (RFS) showing association of high *YAP1* expression with worse patient outcomes only in *TP53* mutant cases. The plots were generated from published data in http://kmplot.com/analysis/.

Supplementary Table 1. Summary of mammary tumours developed in induced mice in Figure 4a.

Tumour name	K8+ *	K5+ **	K8+K5+	K14+ **	K8+K14+	Tumour type ***	Genomic lesion ****
E1	+++	+	+/-	+++	++	Type 1	*Yap1, (Akt3)*
E1-2	++	+/-	-	++	+	Type 1	ND
E1-3	+++	(-)	-	++	+	Type 2	ND
E2	+	+	(-)	++	+	Type 1	*Yap1*
E2-2	++	(-)	-	+	+/-	Type 1	ND
E3	++	+	+/-	+++	++	Type 1	*Yap1, Met*
E4	++	+/-	-	+	+/-	Type 1	*Yap1*
E4-2	+	+/-	(-)	+	+	Type 1	*Yap1*
E5	++	(-)	(-)	+++	++	Type 1	*Yap1*
E6	+	(-)	(-)	+	+	Type 1	*Yap1*
E6-2	+	(-)	(-)	+/-	+/-	Type 1	ND
E6-3	+++	(-)	(-)	++	+	Type 2	ND
E7	+/-	(-)	(-)	+/-	+/-	Type 1	*Met*
E7-2	++	+++	+	++	+	Type 1	*Yap1*
E8	+++	(-)	-	+++	++	Type 2	*Met, Akt3*
E8-2	++++	+/-	(-)	++++	+++	Type 2	*Yap1*
E9	+	+/-	+/-	+/-	+/-	Type 1	*Yap1, Met, Akt3*
E9-2	++	+	(-)	++	++	Type 2	ND
TB208	+	++	+/-	+++	+/-	Type 1	*Yap1*
TB209	+	(-)	(-)	++	+	Type 1	*Yap1*
TB209-2	+	(-)	(-)	+	+	Type 1	*Yap1*
TB239	++	+/-	+/-	+++	++	Type 1	*Yap1*
TB239-2	(-)	(-)	(-)	+/-	(-)	Type 1	*Yap1, Met, (Akt3)*
TB242	+	(-)	(-)	+	+/-	Type 1	*Yap1, (Met), (Akt3)*

Keratin positivity (i.e., % of positive cells) scoring: ++++ > +++ > ++ > + > +/- > (-) > -

*: Although scored positive for K8, Type 1 tumours typically exhibited weaker positivity for K8 than Type 2 tumours.

**: Single K5 or K14 positive cells tend to have stronger K5 or K14 staining than that in $K8^+K5^+$ or $K8^+K14^+$ cells.

***: Type 1: tumours with more spindle cell-like appearance; Type2: tumours with more epithelial cell-like morphology.

****: Based on PCR analyses in Fig. 6; genes in parentheses represent those with elevated gene expression but no detected gene amplification. ND: no data.

Supplementary Reference:

1. Lim, E. et al. Transcriptome analyses of mouse and human mammary cell subpopulations reveal multiple conserved genes and pathways. *Breast Cancer Res* **12**, R21 (2010).
